# MAPK Cascades in Guard Cell Signal Transduction

**DOI:** 10.3389/fpls.2016.00080

**Published:** 2016-02-11

**Authors:** Yuree Lee, Yun Ju Kim, Myung-Hee Kim, June M. Kwak

**Affiliations:** ^1^Center for Plant Aging Research, Institute for Basic ScienceDaegu, South Korea; ^2^Department of New Biology, Daegu Gyeongbuk Institute of Science and TechnologyDaegu, South Korea

**Keywords:** MAPK cascade, stomatal pore, guard cell development, stomatal movement, environment

## Abstract

Guard cells form stomata on the epidermis and continuously respond to endogenous and environmental stimuli to fine-tune the gas exchange and transpirational water loss, processes which involve mitogen-activated protein kinase (MAPK) cascades. MAPKs form three-tiered kinase cascades with MAPK kinases and MAPK kinase kinases, by which signals are transduced to the target proteins. MAPK cascade genes are highly conserved in all eukaryotes, and they play crucial roles in myriad developmental and physiological processes. MAPK cascades function during biotic and abiotic stress responses by linking extracellular signals received by receptors to cytosolic events and gene expression. In this review, we highlight recent findings and insights into MAPK-mediated guard cell signaling, including the specificity of MAPK cascades and the remaining questions.

## Introduction

During photosynthesis, the gas exchange and water transpiration between the plant and the atmosphere take place through the microscopic pores surrounded by guard cells in the epidermis, called stomata (Schroeder et al., 2001). Open stomata help in CO_2_ absorption but also render plants vulnerable to dehydration and pathogen attacks (Schroeder et al., 2001; Nilson and Assmann, 2007; Gudesblat et al., 2009). Precise regulation of stomatal aperture in response to endogenous and environmental stimuli, such as light, CO_2_, temperature, hormones, drought, and pathogens, is crucial for plant growth and survival. To achieve this, guard cells integrate developmental and environmental stimuli into the signaling networks, which include mitogen-activated protein kinase (MAPK) cascades.

Mitogen-activated protein kinase cascades are composed of at least three kinases: MAPK kinase kinases (MAP3Ks), MAPK kinases (MAP2Ks or MKKs), and MAPKs (for which we have adopted MPK). These enzymes are highly conserved in all eukaryotes, and they play crucial roles in diverse developmental and physiological processes (Mapk-Group, 2002; Rodriguez et al., 2010). In general, signals received by receptors are transduced to MAP3Ks, which evoke subsequent phosphorylation events. MAP3Ks are Ser/Thr kinases and phosphorylate MAP2Ks on two Ser/Thr residues (S/T-XXXXX-S/T, X denotes any amino acid). MAP2Ks are dual-specificity protein kinases that phosphorylate MPKs on Thr and/or Tyr residues in the TXY motif. Phosphorylated MPKs transduce the signals to various effector molecules regulating their functional activity, turnover, and localization.

In plants, MAPK pathways function in developmental processes, immune response, abiotic stress responses, and hormonal regulation (Liu et al., 2010; Rodriguez et al., 2010; Moustafa et al., 2014; Arnaud and Hwang, 2015). The multi-functionality of MAPK cascades in cellular signaling is probably due to the fact that MPKs, MAP2Ks, and MAP3Ks are encoded by gene families with large numbers of genes (Mapk-Group, 2002). In the *Arabidopsis* genome, approximately 110 genes encode MAPK cascade components: 20 MPKs, 10 MAP2Ks, and 80 MAP3Ks (Jonak et al., 2002; Mapk-Group, 2002; Menges et al., 2008). MAP3Ks form the largest family of MAPK cascades with three clades: MEKKs (MAP3K1-21), RAF-like (RAF1-48), and ZIK-like (ZIK1-11). The members of these gene families could form different combinatorial MAPK cascades, allowing the cells to deal with diverse stimuli, possibly in a stimulus-specific manner. Identifying functional, stimulus-specific MAPK modules out of thousands of possible combinations has long been a goal, which has begun to be achieved, at least in part, by systematical approaches (Asai et al., 2002; Lee et al., 2008; Umezawa et al., 2013). In addition to the classical three-tiered kinase cascades, there are 10 genes in the *Arabidopsis* genome encoding MAP3K kinases, but their implication on MAPK cascades is still unclear.

In this review, we highlight recent findings for MAPK-mediated guard cell signaling and discuss the specificity of MAPK cascades and questions that await answers.

## MAPK Cascades in ABA-Induced Stomatal Closure

ABA regulates stomatal movements in response to biotic and abiotic stresses. ABA signaling received by the ABA receptor PYR/PYL/RCAR is transduced to activate SNF1-related protein kinase 2 (SnRK2) (Ma et al., 2009; Park et al., 2009; Soon et al., 2012). This initiates subsequent cellular events including the production of reactive oxygen species (ROS) and nitric oxide, elevation of cytosolic Ca^2+^ levels, cytosolic alkalization, activation of anion and calcium channels, and loss of guard cell turgor (Lim et al., 2015). In addition, a MAPK activity has been detected in guard cell protoplasts treated with ABA (Mori and Muto, 1997). A pharmacological study using the MAP2K inhibitor PD98059 in pea epidermal peels has shown inhibition of ABA-induced stomatal closure and ABA-inducible dehydrin gene expression (Burnett et al., 2000). These biochemical and pharmacological studies suggest that MAPK cascades are involved in ABA signaling in guard cells.

Spatial expression patterns of genes provide some hints to their roles where they are expressed. Gene regulation in response to certain stimuli indicates the role of these genes in dealing with the stimuli. A proteomics study of guard cell proteins has shown that MPK4, MPK9, MPK12, and MKK2 proteins are present in *Arabidopsis* guard cells (Zhao et al., 2008). ABA upregulates the expression of *MPK3*, *MPK5*, *MPK7*, *MPK18*, *MPK20*, *MKK9*, *MAP3K1*, *MAP3K10* (*MEKK3*), *MAP3K14*, *MAP3K15*, *MAP3K16*, *MAP3K17*, *MAP3K18*, and *MAP3K19* (Menges et al., 2008; Wang et al., 2011). A cell type-specific transcriptomics analysis has revealed that *MPK4*, *MPK5*, *MPK9*, *MPK11*, *MPK12*, *MPK17*, and *MPK19* are highly expressed relatively in guard cells (Leonhardt et al., 2004; Jammes et al., 2009). Eleven of the 80 MAP3K genes appear to be highly expressed relatively in *Arabidopsis* guard cells: *MAP3K11* (*MEKK4*), *RAF6*, *RAF15*, *RAF17*, *RAF19*, *RAF22*, *RAF29*, *RAF33*, *RAF34*, *RAF40*, and *ZIK4* (Leonhardt et al., 2004). In contrast, *MAP2K* genes seem to be expressed in these cells at very low levels (Leonhardt et al., 2004). Combined analysis of spatial expression patterns and ABA regulation of the MAPK cascade genes could contribute to deciphering specific MAPK cascades involved in ABA signaling in guard cells. Interestingly, antisense suppression of *MPK3* (in a guard cell-specific manner) results in impaired ABA inhibition of stomatal opening and H_2_O_2_-induced stomatal closure. However, ABA-induced stomatal closure and ABA-induced H_2_O_2_ production are not affected (Gudesblat et al., 2007). Lu et al. (2002) have reported that MPK3 is activated by ABA and H_2_O_2_, suggesting its role in ABA signaling. However, *MPK3* expression in guard cells has not been examined by Gudesblat et al. (2007). Thus, it is unclear whether the stomatal phenotype in the antisense of *MPK3* plants is because of the suppression of *MPK3* in guard cells or suppression of other member(s) of the MPK gene family expressed in guard cells which are closely related to *MPK3*.

A cell type-specific functional genomics approach has revealed a high and preferential expression of two MPK genes, *MPK9* and *MPK12* in guard cells (Jammes et al., 2009). *Arabidopsis* mutants with mutations in both *MPK9* and *MPK12* show reduced ABA promotion of stomatal closure, ABA inhibition of stomatal opening, impaired ABA and calcium activation of anion channels, and enhanced transpiration water loss in the leaves (Jammes et al., 2009). MPK12 kinase activity is enhanced by ABA and H_2_O_2_ treatment. MPK9 and MPK12 show functional overlap and act downstream of calcium and upstream of anion channels in ABA signaling (Jammes et al., 2009). Another study has reported that MPK9 and MPK12 function in ABA-induced stomatal closure, whereas MPK3 and MPK6 function in flg22-induced stomatal closure (Montillet et al., 2013). An independent quantitative trait locus analysis of natural accessions of *Arabidopsis* has shown that MPK12 carrying an amino acid substitution causes reduction in water use efficiency (Des Marais et al., 2014). The effect MPK12 has on water use efficiency is not shared by MPK9, unlike the overlapping functions of these enzymes in ROS-mediated stomatal closure in response to ABA. This result suggests that all functions of MPK12 are not redundant (Des Marais et al., 2014). It would be interesting to identify substrates of MPK9 and MPK12 in these cellular processes, which would provide further insights into MPK9- and MPK12-mediated guard cell signaling. Moreover, which specific MAP2K and MAP3K participate in the complete cascade involving MPK9 and MPK12 in ROS-mediated ABA signaling and in water use efficiency remains to be established. Further studies are required to determine the detailed mechanism by which these MAP kinases regulate anion channel activity and stomatal closure.

Besides MPK9 and MPK12, the MKK1–MPK6 module positively regulates *CATALASE1* expression and ABA-induced H_2_O_2_ production in guard cells (Xing et al., 2008). However, reduced ROS production in single *mpk6* mutants does not impair the stomatal closure in response to ABA (Montillet et al., 2013). Hydrogen peroxide activates the ANP1 (MAP3K1) activity, which subsequently leads to phosphorylation and activation of MPK3 and MPK6 (Kovtun et al., 2000). However, it is not clear whether ANP1-initiated MAPK cascades act in ROS-mediated guard cell signaling.

In guard cells, the soluble PYR/PYL/RCAR ABA receptors bind ABA and interact with the inhibitory protein phosphatases 2C (PP2Cs), which results in release of the active form of SnRK2 protein kinases (Ma et al., 2009; Park et al., 2009; Soon et al., 2012). In addition to other SnRK2.6 substrates, Ser13 and Ser174 on NADPH oxidase RbohF are phosphorylated by SnRK2.6 (Sirichandra et al., 2009). Interestingly, a phosphoproteomics study has shown that MPK1 and MPK2, which are activated by ABA and H_2_O_2_ (Ortiz-Masia et al., 2007), are phosphorylated in a SnRK2-dependent manner (Umezawa et al., 2013). ABA dependent phosphorylation of MPK1 and MPK2 is reduced in *snrk2.2/2.3/2.6* triple mutants, suggesting that SnRK2 promotes the activation of MPK1 and MPK2 by ABA (Umezawa et al., 2013). In addition, MPK1 and MPK2 have been identified as a part of ABA-activated MAPK modules, MAP3K17/18-MAP2K3-MPK1/2/7/14 (Danquah et al., 2015). Interestingly, MAP3K18 is expressed in guard cells and has been recently shown to be regulated by the ABI1 protein phosphatase, suggesting its role in ABA signaling in guard cells. Indeed, *map3k18* mutants show an increase in stomatal aperture under normal growth conditions as well as in response to ABA treatment, compared with the wild-type (Mitula et al., 2015). However, *map3k18* mutants have significant reduction in the stomatal index, which could affect stomatal apertures (Bussis et al., 2006; Franks et al., 2015). Thus further studies are required to determine whether and how ABA activation of MAP3K18 leads to stomatal closure.

## MAPK Cascades in Immune Response in Guard Cells

Open stomata make the plants vulnerable to microbial invasion and closing mechanisms have been evolved to prevent stomatal pores from being used as a gate for pathogens by rapidly closing the pores upon pathogen recognition (Melotto et al., 2006). Pathogen-induced stomatal closure is triggered by pathogen/microbe-associated molecular patterns (PAMPs or MAMPs). Various molecules are involved in this response: oligogalacturonic acid, chitosan, flg22 (a peptide derived from bacterial flagellin), and lipopolysaccharide (Lee et al., 1999; Klusener et al., 2002; Melotto et al., 2006; Gudesblat et al., 2009). PAMPs registered by the host pattern-recognition receptors initiate a variety of defense responses. These responses include the production of ROS and nitric oxide, elevation of cytosolic Ca^2+^ levels, activation of salicylic acid signaling pathway, synthesis of ethylene, and stomatal closure (Arnaud and Hwang, 2015).

Mitogen-activated protein kinase cascades confer another line of defense mechanism by regulating the activation of defense genes, synthesis of antimicrobial metabolites, and hypersensitive response-like cell death (Zhang and Klessig, 2001; Asai et al., 2002; Pedley and Martin, 2005). Recent studies have found that major regulators of innate immune response such as MPK3, MPK4, and MPK6 also participate in the stomatal defense. Antisense suppression of *MPK3* in a guard cell-specific manner causes impaired stomatal closure in response to bacteria, lipopolysaccharide, and stomatal inhibiting factor from bacterial phytopathogen *Xanthomonas campestris* pv *campestris* (Gudesblat et al., 2009). MPK3 and MPK6 activate guard cell-specific lipoxygenase, LOX1 that contributes to the synthesis of a large number of oxylipins thereby closing stomatal pores in response to both bacteria and flg22 (Montillet et al., 2013). MPK3- and MPK6-mediated stomatal closure in response to pathogen infection is independent of ABA, as *mpk3* and *mpk6* single mutants show normal stomatal closure in response to this compound (Montillet et al., 2013).

The role of MPK4 appears to be distinct from those of MPK3 and MPK6 in stomatal immune response. *mpk4* mutant plants display enhanced resistance to the bacterial pathogen *Pseudomonas syringae* pv *tomato (Pst)* (Petersen et al., 2000). Transgenic plants that harbor a constitutively active form of *MPK4* (*CA-MPK4*) have compromised disease resistance and are more susceptible to *Pst* infection than normal plants. However, the resistance of *CA-MPK4* plants is not weakened when bacteria are injected directly into the leaf apoplast. As *MPK4* is expressed in guard cells (Petersen et al., 2000), it has been suggested that it mediates stomata-based defense against bacterial entry (Berriri et al., 2012). However, the stomatal closure in response to *Pst* DC3000 and flg22 in *CA-MPK4* transgenic plants and in wild-type plants showed no difference. This implies that the defense function of MPK4 is not linked to the regulation of stomatal apertures (Berriri et al., 2012). Interestingly, *MPK4* homologs in *Nicotiana attenuata* and *N. tabacum* positively regulate stomatal closure in response to biotic and abiotic stresses (Gomi et al., 2005; Marten et al., 2008; Hettenhausen et al., 2012). These differences might be species specific, and further investigation is necessary to clarify the mechanism by which MPK4 enzymes regulate stomatal closure.

**FIGURE 1:**
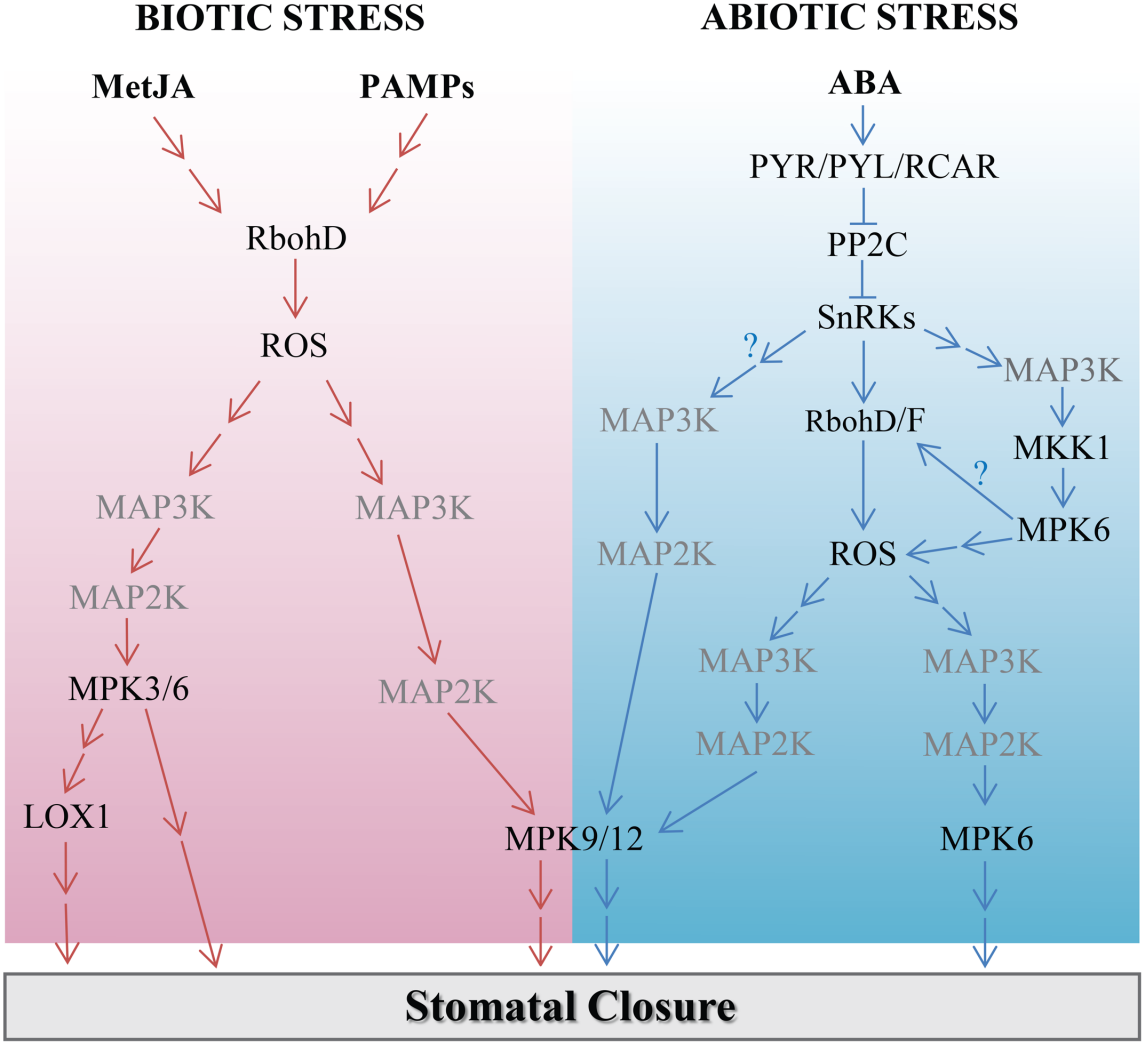
**Working model of mitogen-activated protein kinase (MAPK) cascades regulating stomatal movement in response to biotic and abiotic stimuli.** Unidentified MAPK components are presented in gray color. MAPK cascades waiting to be revealed are indicated in arrows with a question mark.

Two *Arabidopsis* guard cell MPK genes, *MPK9* and *MPK12*, regulate stomatal apertures in response to biotic stimuli. *mpk9mpk12* double mutants are highly susceptible to *Pst* when the pathogen is sprayed on the leaves (Jammes et al., 2011). Moreover, in *mpk9mpk12* double mutants, but not in *mpk9* or *mpk12* single mutants, the methyl jasmonate-induced stomatal closure is impaired. This indicates a functional redundancy of these two genes in the methyl jasmonate signaling in guard cells (Khokon et al., 2015). Anion channel activation by methyl jasmonate is impaired in *mpk9mpk12* double mutants, suggesting that MPK9 and MPK12 act upstream of anion channels during stomatal closure (Khokon et al., 2015). Two other studies have shown that yeast elicitor- and chitosan-induced stomatal closure are defective in *mpk9mpk12* double mutants (Salam et al., 2012, 2013). Interestingly, flg22-induced stomatal closure is normal in these double mutants (Montillet et al., 2013). Overall, these studies show that ABA, methyl jasmonate, and biotic stimuli converge on MPK9 and MPK12 in guard cells (**Figure 1**). Identification of target proteins of MPK9 and MPK12 would broaden our understanding of MAPK-mediated signaling in guard cells.

## MAPKS in Stomatal Development

Not only guard cell signaling but also the development of stomata, including stomatal density (number of stomata per mm^2^ of leaf) and stomatal index (ratio of stomata to epidermal cells), are tightly regulated in response to various developmental and environmental stimuli (Lake et al., 2001). For instance, high CO_2_ concentration and low intensity light decrease both stomatal index and density, whereas high intensity light increases these values (Schoch et al., 1980; Lake et al., 2001; Chater et al., 2015).

Stomatal development is accomplished by a series of asymmetric cell divisions, differentiation, and then a symmetric division that generates a pair of guard cells. These processes require a ligand–receptor module, MAPK signaling cascades, and nuclear transcription factors (Lau and Bergmann, 2012). In these developmental processes, MAPK pathways integrate the intrinsic signal from the ligand–receptor module and transduce the signal to the transcriptional factors to allow stomatal development and patterning.

Involvement of MAPK signaling in stomatal development has been examined in a study of the MAP3K YODA (Bergmann et al., 2004). *yoda* mutants have a large number of clustered guard cells, whereas constitutive activation of YODA results in stomataless phenotypes. Stomatal clusters in *yoda* mutants, consisting of paired guard cells, are different from the clusters of unpaired guard cells formed by repeated divisions of the guard mother cells in *four lips (flp)* mutants (Yang and Sack, 1995). This suggests that increased density of guard cells in *yoda* mutants is due to the defective asymmetric cell division before the differentiation into guard mother cells.

The downstream cascades of YODA include MKK4/MKK5 and MPK3/MPK6 (Wang et al., 2007). MKK4/MKK5 and MPK3/MPK6 act as negative regulators of stomatal patterning by controlling asymmetric cell divisions and differentiation of stomatal cells. *mkk4mkk5* or *mpk3mpk6* knockdown mutants have clustered guard cells. Stomatal differentiation is suppressed in transgenic plants expressing constitutively active forms of MKK4 and MKK5 (Wang et al., 2007). In addition, stress-associated MKK7 and MKK9 have functions overlapping with those of MKK4 and MKK5, thus activating MPK3/MPK6 and preventing meristemoids from entering the stomatal lineage (Lampard et al., 2009). At later stages of stomatal development, however, YODA–MKK7/9 modules promote stomatal development in guard mother cells in a cell-type specific manner. In addition to YODA, a recent study shows ABA-activated MAP3K18 acts as a positive regulator of stomatal development (Mitula et al., 2015). These results suggest that stomatal development regulated by MAPK cascades is not a simple, linear process. The complex regulatory mechanisms of this process are yet to be elucidated.

Recent studies have revealed a broad range of downstream targets of MAPK cascades in stomatal development. Breaking of Asymmetry in the Stomatal Lineage (BASL) is one of them, and phosphorylation of BASL by MPK3/MPK6 is required for its polarized localization (Zhang et al., 2015). *basl* mutants lack asymmetric divisions, resulting in daughter cells with similar size and identity (Dong et al., 2009). BASL localizes initially in the nucleus and begins to accumulate in a cortical crescent before the asymmetric cell division. This polarized localization pattern is inherited only by a larger daughter cell, which becomes a non-stomatal lineage cell. BASL has five serine residues phosphorylated by MPK3 and MPK6 (Zhang et al., 2015). When these residues are modified to Ala, BASL is sequestered in the nucleus and the stomatal defects of *basl* mutants do not rescued in complementation lines (Zhang et al., 2015). This observation suggests that MPK3/6 -mediated phosphorylation of BASL is required for the transfer of this protein to the cortical pool; this localization is critical for BASL function in a polarity module. Interestingly, phosphorylated BASL strongly interacts with YODA to recruit it into the cell cortex (Zhang et al., 2015). These results indicate that a biased spatial regulation of BASL by the MAPK cascades through the positive feedback loop determines the fate of daughter cells during cell division.

Transcription factors are other downstream targets of MAPK cascades in stomatal development. Three basic helix–loop–helix (bHLH) transcription factors, SPEECHLESS (SPCH), MUTE, and FAMA, are necessary for three critical steps of stomatal development: initiation by SPCH, meristemoid differentiation by MUTE, and final guard cell differentiation by FAMA (Ohashi-Ito and Bergmann, 2006; MacAlister et al., 2007; Pillitteri et al., 2007). *In vitro* phosphorylation analysis has shown that SPCH is a direct phosphorylation target of MPK3 and MPK6. Constitutive expression of the mutated *SPCH* without MAPK target domains induces the formation of excess stomata (Lampard et al., 2008). Genetic analyses have revealed that MPK3 and MPK6 negatively regulate SPCH function by altering persistence of SPCH (Lampard et al., 2008). Moreover, MUTE and the MYB transcription factor MYB88 are phosphorylated by MPK6 and MPK4 *in vitro*, respectively (Popescu et al., 2009). These findings indicate that MPK3, MPK4, and MPK6 have targets in both the nucleus and cytosol. Thus, it would be interesting to investigate how the localization of these MAPK proteins is controlled upon different stimuli.

## MAPK Phosphatases in Guard Cell Signaling

Mitogen-activated protein kinase cascades activated by sequential events of phosphorylation can be reversed by dephosphorylation. This process is mediated by protein phosphatases including Tyr-specific phosphatases, Ser/Thr phosphatases, and dual-specificity phosphatases (Brock et al., 2010). Regulation of the duration of MAPK activities has been suggested as one of the mechanisms to render specificity and fine-tune the MAPK-mediated signal transduction (Ebisuya et al., 2005; Murphy and Blenis, 2006).

Mitogen-activated protein kinase phosphatases (MKPs) belong to a group of specialized dual-specificity phosphatases, which dephosphorylate both Ser/Thr and Tyr residues, and act as negative regulators of MAPKs (Camps et al., 2000). The *Arabidopsis* genome encodes five potential MKPs (MKP1, MKP2, DsPTP1, PHS1, and IBR5; Kerk et al., 2002). They regulate the activities of components of MAPK cascades in various stress signaling pathways (Ulm et al., 2002; Lee and Ellis, 2007; Schweighofer et al., 2007; Bartels et al., 2009; Lee et al., 2009; Brock et al., 2010). Indole-3-Butyric acid-Response 5 (IBR5) dephosphorylates MPK12 through direct interaction and functions in auxin and ABA signaling (Lee et al., 2009). MKP1 and Protein Tyrosine Phosphatase 1 interact with MPK6 and negatively regulate MPK3/MPK6-mediated stress responses (Ulm et al., 2002; Bartels et al., 2009). MKP2 dephosphorylates *Arabidopsis* MPK3 and MPK6 *in vitro*, and *mkp2*-knockdown plants exhibit enhanced sensitivity to ozone stress (Lee and Ellis, 2007). However, it remains unclear whether those enzymes are also involved in stomatal movement.

A recent study of *OsIBR5*, the closest homolog of *AtIBR5*, has supplied a clue to the involvement of MKP in stomatal movements (Li et al., 2012). Tobacco plants overexpressing *OsIBR5* show impaired stomatal closure in response to drought and ABA and are hypersensitive to drought and oxidative stresses. These suggest that OsIBR5 has a role as a negative regulator in ABA- and drought-mediated response. Interestingly, *OsIBR5* is upregulated in response to ABA and H_2_O_2_ in rice seedlings, suggesting a regulatory feedback mechanism between MAPKs and MKPs. OsIBR5 interacts with two tobacco MAPKs: SIPK and WIPK, and the drought-inducible kinase activity of WIPK is suppressed in *OsIBR5*-overexpressing plants. Though further studies are required to obtain detailed physiological functions of the OsIBR5–MAPK complexes in rice, these results clearly show the involvement of MKP in the regulation of stomatal apertures.

Besides the MKPs, PP2Cs utilize phosphorylated MAPKs as substrates. PP2C-type phosphatase AP2C1 inactivates the stress-responsive MPKs, MPK4, and MPK6 (Schweighofer et al., 2007). PP2C5 directly interacts with MPK3, MPK4, and MPK6 and inhibits their kinase activities induced by ABA (Brock et al., 2010). Both *pp2c5* and *ap2c1* single mutants show increased stomatal apertures. This phenotype is clearly pronounced in the *pp2c5ap2c1* double mutants, suggesting functional redundancy of the two genes (Brock et al., 2010). Interestingly, these double mutants do not affect the response to *Pst* DC3000 infection, indicating that a different regulatory mechanism is involved in pathogen-induced stomatal closure.

## Perspectives and Concluding Remarks

It is clear that MAPK cascades play important roles in the fine-tuning of complex cellular signaling networks in response to biotic and abiotic stimuli in plants (**Figure 1**). Although the MPK, MAP2K, and MAP3K gene families contain a large number of genes, the functions of only a handful of these genes have been identified in cell signaling and/or development in plants (**Figure 1**). This is probably largely due to the high level of functional redundancy of these genes. Therefore, it would be advisable to use biochemical and genetic approaches that can address the issue, including artificial miRNAs that simultaneously knockdown homologous genes with potential overlapping functions (Hauser et al., 2013), use of constitutively active forms of MAPKs (Berriri et al., 2012), and simultaneous targeting of homologous genes using the CRISPR/Cas9 system (Woo et al., 2015). A cell type-specific phosphoproteomics approach using genetic mutants could help to complete MAPK cascades; it could also provide the means to the classification of components of MAPK cascades mediating stimulus-specific response in guard cells.

## Author Contributions

YL and JK conceived the manuscript, and all authors contributed to writing the manuscript. All authors approved the final manuscript.

## Conflict of Interest Statement

The authors declare that the research was conducted in the absence of any commercial or financial relationships that could be construed as a potential conflict of interest.
